# Diagnostic performance of attenuation imaging versus controlled attenuation parameter for hepatic steatosis with MRI-based proton density fat fraction as the reference standard: a prospective multicenter study

**DOI:** 10.1007/s00535-025-02224-0

**Published:** 2025-02-24

**Authors:** Takashi Nishimura, Toshifumi Tada, Tomoyuki Akita, Reiichiro Kondo, Yasuaki Suzuki, Kento Imajo, Shigehiro Kokubu, Tamami Abe, Hidekatsu Kuroda, Masashi Hirooka, Yoichi Hiasa, Asako Nogami, Atsushi Nakajima, Sadanobu Ogawa, Hidenori Toyoda, Satoshi Oeda, Hirokazu Takahashi, Yuichiro Eguchi, Katsutoshi Sugimoto, Hirohisa Yano, Junko Tanaka, Fuminori Moriyasu, Masayoshi Kage, Takashi Kumada, Hiroko Iijima

**Affiliations:** 1 Division of Hepatobiliary and Pancreatic Disease, Department of Gastroenterology, Hyogo Medical University, 1-1 Mukogawa-cho, Nishinomiya, Japan; 2 Ultrasound Imaging Center, Hyogo Medical University Hospital, Nishinomiya, Japan; 3 Department of Internal Medicine, Japanease Red Cross Himeji Hospital, Himeji, Japan; 4 Department of Epidemiology, Infectious Disease Control, and Prevention, Hiroshima University Institute of Biomedical and Health Sciences, Hiroshima, Japan; 5 Department of Pathology, Kurume University School of Medicine, Kurume, Fukuoka Japan; 6 Department of Gastroenterology, Nayoro City General Hospital, Nayoro, Japan; 7 Department of Gastroenterology, Yokohama City University Graduate School of Medicine, Yokohama, Japan; 8 Department of Gastroenterology, Shin-Yurigaoka General Hospital, Kawasaki, Japan; 9 Division of Hepatology, Department of Internal Medicine, Iwate Medical University, Yahaba, Japan; 10 Department of Gastroenterology and Metabology, Ehime University Graduate School of Medicine, Toon, Japan; 11 Department of Clinical Research, Ogaki Municipal Hospital, Ogaki, Japan; 12 Department of Gastroenterology and Hepatology, Ogaki Municipal Hospital, Ogaki, Japan; 13 Liver Center, Saga Medical School, Saga University, Saga, Japan; 14 Department of Laboratory Medicine, Saga University Hospital, Saga, Japan; 15 Department of Gastroenterology and Hepatology, Tokyo Medical University, Tokyo, Japan; 16 Department of Gastroenterology and Hepatology, International University of Health and Welfare, Sanno Hospital, Tokyo, Japan; 17 Center for Innovative Cancer Therapy, Kurume University Research, Kurume, Japan; 18 Department of Nursing, Gifu Kyoritsu University, Ogaki, Japan

**Keywords:** Attenuation imaging (ATI), Controlled attenuation parameter (CAP), Magnetic resonance imaging-based proton density fat fraction (MRIPDFF), 2-dimensional share wave elastography (2D-SWE), Hepatic steatosis

## Abstract

**Background:**

Attenuation Imaging (ATI) and controlled attenuation parameter (CAP) are non-invasive ultrasound-based methods for diagnosing hepatic steatosis. However, reports on the clinical usefulness of ATI are limited. We aimed to compare the ability of ATI and CAP to diagnose hepatic steatosis with magnetic resonance imaging–based proton density fat fraction (MRI-PDFF) as the reference standard.

**Methods:**

We performed a prospective multicenter study of 562 patients with chronic liver disease who underwent ATI, CAP, and MRI-PDFF. Patients with skin-to-liver capsule distance (SCD) ≤ 25 mm underwent CAP with an M probe; those with SCD > 25 mm underwent CAP with an XL probe. MRI-PDFF was used as the reference standard: S0 corresponds to MRI-PDFF < 5.2%, S1 to 5.2% ≤ MRI-PDFF < 11.3%, S2 to 11.3% ≤ MRI-PDFF < 17.1%, and S3 to MRI-PDFF ≥ 17.1%.

**Results:**

The correlation coefficients for ATI and MRI-PDFF stratified by body mass index (< 30, ≥ 30 kg/m^2^), SCD (< 25, ≥ 25 mm), 2-dimensional share wave elastography (< 1.8 m/s), fibrosis-4 index (≤ 2.67), albumin–bilirubin score (< − 2.60) and type IV collagen 7 s (< 5.0 ng/ml) were significantly higher than those for CAP and MRI-PDFF. Areas under the receiver operating characteristics (95% CI) for ATI and CAP were 0.895 (0.869–0.922) and 0.845 (0.809–0.881) for ≥ S1 steatosis, 0.944 (0.926–0.963) and 0.881(0.852–0.910) for ≥ S2 steatosis, and 0.928 (95% CI 0.906–0.950) and 0.860 (95% CI 0.829–0.890) for S3 steatosis. ATI had higher diagnostic performance for all hepatic steatosis grades than CAP.

**Conclusions:**

ATI is a more useful non-invasive method for diagnosing hepatic steatosis than CAP.

**Supplementary Information:**

The online version contains supplementary material available at 10.1007/s00535-025-02224-0.

## Introduction

Recently, a multi-society Delphi conference proposed a change in the name NAFLD to metabolic dysfunction–associated steatotic liver disease (MASLD) [1]. To diagnose MASLD in clinical settings, the degree of hepatic steatosis and fibrosis is important. Hepatic fibrosis is correlated with long-term overall mortality, liver transplantation, and liver-related events such as HCC and liver cirrhosis [2]. Hepatic steatosis is associated with extrahepatic complications [3, 4]. In addition, in patients with chronic hepatitis C, including those with eradication after direct-acting antiviral (DAA) therapy, steatosis is associated with fibrosis [5], especially MASLD at 24 weeks of sustained virological response in elderly patients with chronic hepatitis C is a risk factor for HCC development [6]. In chronic hepatitis B, amelioration of hepatic steatosis is associated with the development of HCC [7]. On the other hand, lenvatinib, a tyrosine kinase inhibitor used to treat advanced hepatocellular carcinoma (HCC), might be a suitable agent for the advanced HCC in patients with MASLD [8]. Therefore, the diagnosis of hepatic steatosis is very important regardless of etiology.

MRI-PDFF is an MRI-based method for quantitatively assessing hepatic steatosis. Several manufacturers of MRI scanners offer it as an option. MRI-PDFF correlates with histologically determined steatosis grade [9, 10].

Several studies have evaluated attenuation measured by ultrasound to assess hepatic steatosis [11–16]. In particular, controlled attenuation parameter (CAP) measured with vibration-controlled transient elastography (VCTE) (FibroScan; Echosens, Paris, France) is a type of attenuation imaging that is used widely worldwide. Attenuation imaging (ATI) has been developed as a non-invasive method for diagnosing the degree of hepatic steatosis by Canon Medical Systems (Otawara, Japan) [12]. The diagnostic performance of ATI in hepatic steatosis with MRI-PDFF as the reference standard has been reported [17]. However, a prospective multicenter cohort study of ATI for diagnosing hepatic steatosis has never been performed.

The aim of this prospective, multicenter study was to investigate the performance of ATI in diagnosing hepatic steatosis with MRI-PDFF as the reference standard and to compare its diagnostic ability with CAP for hepatic steatosis.

## Methods

### Subjects

**Fig. 1:**
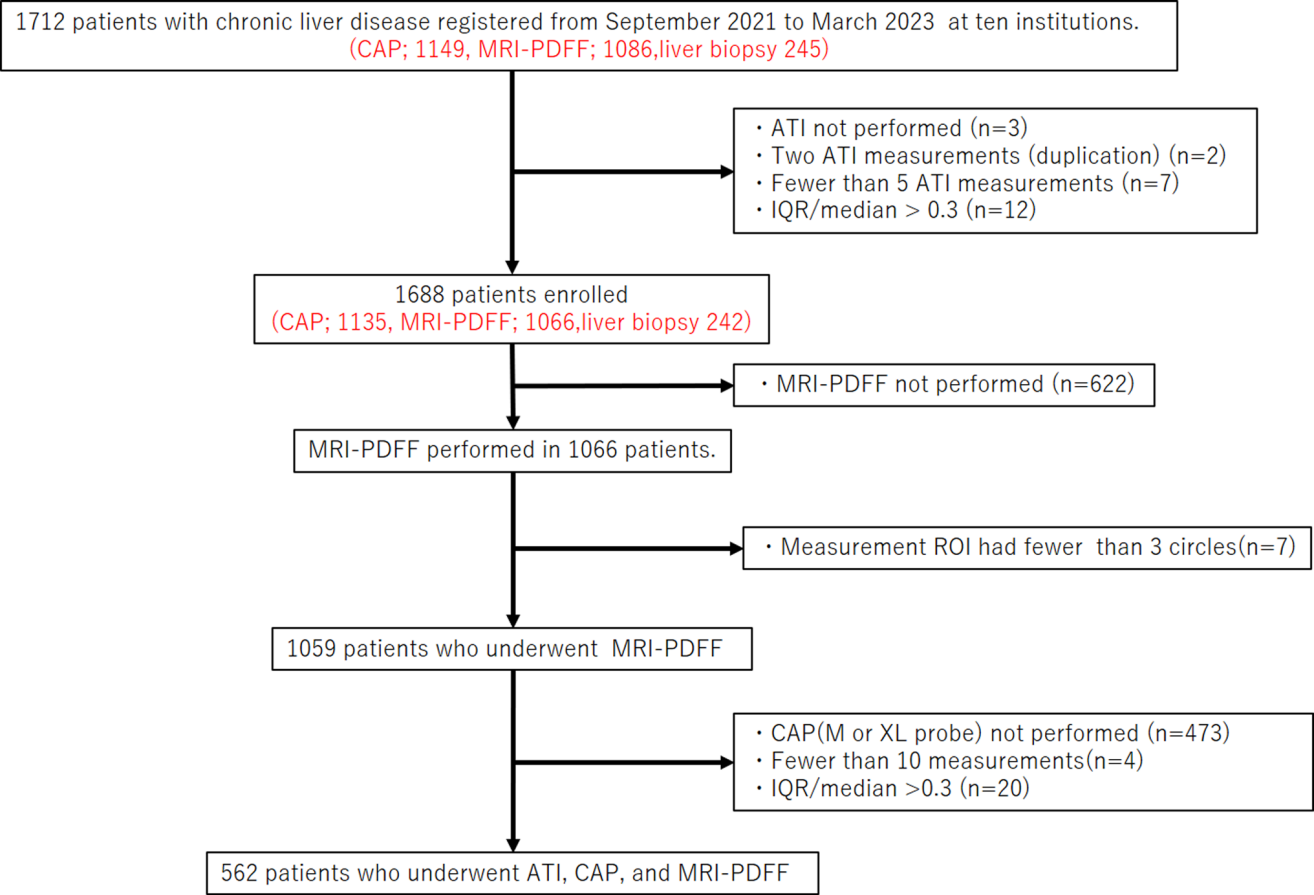
Flowchart of the patient enrollment process. *ATI* attenuation imaging; *CAP* controlled attenuation parameter; *IQR/median* interquartile range/median; *MRI-PDFF* magnetic resonance imaging-based proton density fat fraction; *ROI* region of interest

This study was prospective multicenter cohort conducted in Japan. This study protocol is shown in Fig. 1. Patients were registered from 10 institutions: Nayoro City General Hospital, Iwate Medical University Hospital, Tokyo Medical University Hospital, Shin-Yurigaoka General Hospital, Yokohama City University Hospital, Ogaki Municipal Hospital, Hyogo Medical University Hospital, Japanese Red Cross Society Himeji Hospital, Ehime University Hospital, and Saga University Hospital. This study was approved by Hyogo Medical University and the local research ethics committee of each center. There were 1,712 patients with chronic liver disease enrolled between September 2021 and March 2023. Of these, 562 patients who underwent ATI, CAP, and MRI-PDFF were included in the analysis. Chronic hepatitis B and chronic hepatitis C were defined as positivity for hepatitis B surface antigen and hepatitis C virus antibody, respectively. MASLD and alcohol-associated liver disease (ALD) were diagnosed on the basis of the Eurpopean Association for the Study of the Liver guidelines [1]. Autoimmune hepatitis (AIH) and primary biliary cholangitis (PBC) were diagnosed on the basis of serology, histopathology, or both. All patients provided written informed consent. This study was conducted in accordance with the ethical principles of the 2013 Declaration of Helsinki and registered in the UMIN Clinical Trials Registry (UMIN000048672).

### ATI and 2D-SWE

ATI was performed with the Aplio i800 ultrasound device (Canon Medical Systems) and the PVI-475BX convex probe by expert operators at each institution. The principle of ATI has previously described [12]. Acquisitions were obtained in the right lobe of the liver through intercostal spaces, with the patient lying in the supine position. The attenuation coefficient was measured at fixed frequencies (ATI-Penetration). In the ATI measurement mode, the B-mode image and ATI mode image were expressed side-by-side (Supplementary Fig. 1). A measurement region of interest (ROI) (4 cm × 3 cm) was placed in the ATI 2D color-mapping image, in which the colors indicated the degree of ultrasound attenuation. Vessels and strong artifacts were automatically filtered out. ATI values were measured five times and defined as the median of five measurement values. To evaluate measurement reliability, the interquartile range (IQR)/median (IQR/median) of five ATI measurements was evaluated. The *R*^2^ value, which is related to the determination coefficient when the slope is linearly fitted, was also assessed. The inclusion criteria of ATI results were (1) IQR/median < 0.3 over five measurements, and (2) *R*^2≥ ^0.7 for each of five measurements.

Similar to ATI measurement, 2D shear wave elastography (2D-SWE) was also performed with the same probe. Acquisitions were obtained in the right lobe of the liver through intercostal spaces, with the patient lying in the supine position. A circular measurement ROI with a 1 cm diameter was placed in the 2D SWE mapping ROI (4 cm × 3 cm). The system automatically filtered out values with a low-quality area. The quality parameter was shown in the propagation map, which was displayed as parallel lines with constant intervals between the lines. The measurement ROI was placed in a reliable area. SWE values were defined as the median of five measurements. SWE values with IQR/median > 0.15 over five measurements were excluded [18].

### CAP and VCTE

CAP and VCTE have been performed within 1 month of ATI measurement. Experienced operators at each institution assessed CAP and VCTE in the right liver lobe using a FibroScan^®^ 430, 430 Mini, or 430 Premier. CAP and VCTE was measured after a fast of at least 6 h using the M or XL probe on the same day as ATI measurement. After we measured the skin-to-liver capsule distance (SCD) using the ultrasound B-mode, patients with SCD ≤ 25 mm were evaluated using the M probe. Patients with SCD > 25 mm were evaluated using the XL probe [11]. CAP measurements were carried out using FibroScan until 10 valid measurements were obtained for each patient. Median values were used to quantify hepatic steatosis. In patients with 10 valid CAP and VCTE measurements, IQR/median > 30% was defined as unreliable [11, 19]. Patients with fewer than 10 valid CAP and VCTE measurements or unreliable CAP and VCTE values were excluded from this study. VCTE cutoff values based on the degree of hepatic fibrosis were previously described [20].

### MRI-PDFF

MRI-PDFF have been performed within 1 month of ATI. MRI was performed using a 3 T system (Ingenia: Philips Healthcare, Best, Netherlands; Discovery MR750w 3.0 T: GE Healthcare, Waukesha, WI; or Skyra: Siemens Healthcare, Munich, Germany). Imaging conditions for each manufacturer are shown in Supplementary Table 1. Three measurement ROIs with 2 cm × 2 cm circles were placed in the right lobe on the PDFF parametric maps, avoiding vessels, bile ducts, lesions, and artifacts (Supplementary Fig. 1).

The median MRI-PDFF value of three measurements was used. Steatosis grade was defined as grade 0 (S0) with MRI-PDFF < 5.2%, grade 1 (S1) with 5.2% ≤ MRI-PDFF < 11.3%, grade 2 (S2) with 11.3% ≤ MRI-PDFF < 17.1%, or grade 3 (S3) with MRI-PDFF ≥ 17.1% [20]. S0, S1, S2, and S3 correspond to histologic hepatic steatosis grades of < 5%, 5–33%, 34–66%, and > 66%, respectively [21].

### Clinical and laboratory data

Patient age, sex, height, weight, etiology of liver disease, alcohol intake status, and diabetes mellitus status were recorded within 1 month of ATI measurement. Laboratory data, which included asparate aminotransferase (AST), alanine aminotransferase (ALT), platelet count, fibrosis-4 index (Fib-4 index) [22], gamma-glutamyl transpeptidase (γ-GT), albumin, total bilirubin, albumin-bilirubin score (ALBI score) [23], and type IV collagen 7S [24], were obtained within 1 month of ATI measurement.

### Statistical analysis

Continuous variables are expressed as medians. The Mann–Whitney *U* test and Kruskal–Wallis test with Holm correction were used to assess continuous variables. The cutoff values for body mass index(BMI), SCD, 2D-SWE, FIB-4 index, ALBI score, and type IV collagen 7 s were 30 kg/m^2^ [25], 25 mm [26], 1.8 m/s [27], 2.67 [22], − 2.60 [23], and 5.0 ng/ml [24], respectively. A normal probability plot was used to assess normality of MRI-PDFF, ATI, and CAP values. If the values were not normally distributed, they were transformed to achieve a normal distribution. Relationships between ATI and MRI-PDFF and between ATI and CAP were determined with Pearson’s correlation coefficient (CC), which was classified as minimal (|*r*|< 0.2), weak (|*r*|. 0.2–0.4), moderate (|*r*|. 0.4–0.7), or strong (|*r*|> 0.7) [28]. The diagnostic performance of ATI and CAP (M/XL) with MRI-PDFF as the reference standard was estimated using AUROC analysis. Statistical tests for differences in correlations were performed using the cocor package in R version 4.2.2. Normal probability plots for ATI, CAP, and MRI-PDFF were generated using JMP Pro 15 (SAS Institute, Cary, NC). All authors had access to the study data. All authors reviewed and approved the final manuscript.

## Results

### Patient characteristics

The baseline characteristics of the study patients are shown in Table 1. Patients consisted of 237 (42.2%) women and 325 (57.8%) men. Median age was 59 years. Median BMI was 25.7 kg/m^2^. There were 32 (5.7%) patients with hepatitis B virus(HBV) infection, 33 (5.9%) with hepatitis C virus(HCV) infection, 5 (0.9%) with HBV and HCV coinfection, 344 (61.2%) with metabolic associated steatotic liver disease(MASLD), 11 (2.0%) with autoimmune hepatitis (AIH), 17 (3.0%) with primary biliary cholangitis (PBC), 6 (1.1%) with AIH and PBC, 58 (10.3%) with ALD, 10 (1.8%) with cryptogenic hepatitis, and 46 (8.2%) with another type of hepatitis. Median ATI, CAP, MRI-PDFF, and 2D-SWE values were 0.67 dB/cm/MHz, 275 dB/m, 8.2%, and 1.32 m/s, respectively.

**Table 1:** Characteristics of the study patients (*n* = 562)

Characteristic		Values
Age	Years	59 (49–70)
Sex*	Female	237 (42.2)
	Male	325 (57.8)
BMI	kg/m^2^	25.7 (22.5–29.6)
SCD	mm	18 (16–21)
Alcohol intake*	Present	112 (19.9)
	Absent	450 (80.1)
Diabetes mellitus*	Present	171 (30.4)
	Absent	391 (69.6)
AST	U/L	30 (22–48)
ALT	U/L	34 (21–66)
Platelet count	10^4^ μL	21.5 (16.8–26.1)
FIB-4 index		1.47 (0.92–2.46)
Gamma GT	U/L	47 (29–99)
Albumin	g/dL	4.3 (4.0–4.5)
Total bilirubin	mg/dL	0.8 (0.6–1.1)
ALBI score		− 2.94 (− 3.11 to 2.69)
Type VI collagen 7S	ng/ml	3.8 (3.2–5.1)
ATI	dB/cm/MHz	0.67 (0.57–0.80)
CAP	dB/m	275 (228–316)
MRI-PDFF	%	8.2 (4.0–17.3)
2D-SWE	m/s	1.32 (1.23–1.54)
Etiology of liver disease*	HBV	32 (5.7)
	HCV	33 (5.9)
	HBV and HCV	5 (0.9)
	MASLD	344 (61.2)
	AIH	11 (2.0)
	PBC	17 (3.0)
	AIH and PBC	6 (1.1)
	ALD	58 (10.3)
	Cryptogenic	10 (1.8)
	Other	46 (8.2)

Continuous variables are presented as medians (interquartile range) unless otherwise indicated

*AIH* autoimmune hepatitis; *ALBI* albumin-bilirubin; *ALD* alcohol-associated liver disease; *ALT* alanine aminotransferase; *AST* aspartate aminotransferase; *ATI* attenuation imaging; *BMI* body mass index; *CAP* controlled attenuation parameter; *2D-SWE* 2-dimensional shear wave elastography; *FIB-4* fibrosis-4; *γ-GT* gamma-glutamyl transpeptidase; *HBV* hepatitis B virus; *HCV* hepatitis C virus; *MRI-PDFF* magnetic resonance imaging-based proton density fat fraction; *MASLD* metabolic dysfunction-associated steatotic liver disease; non-alcoholic fatty liver disease; *PBC* primary biliary cholangitis; *SCD* skin capsular distance

*Data are numbers of participants, with percentages in parentheses

### Normal probability plots of MRI-PDFF, ATI, and CAP values

Normal probability plots were used to evaluate the distribution of MRI-PDFF, ATI, and CAP values. ATI and CAP values were distributed normally, but MRI-PDFF values were not (Supplementary Fig. 2). Therefore, MRI-PDFF values were log-transformed (log MRI-PDFF) to achieve a normal distribution.

### Correlations between ATI, CAP, and MRI-PDFF

**Fig. 2:**
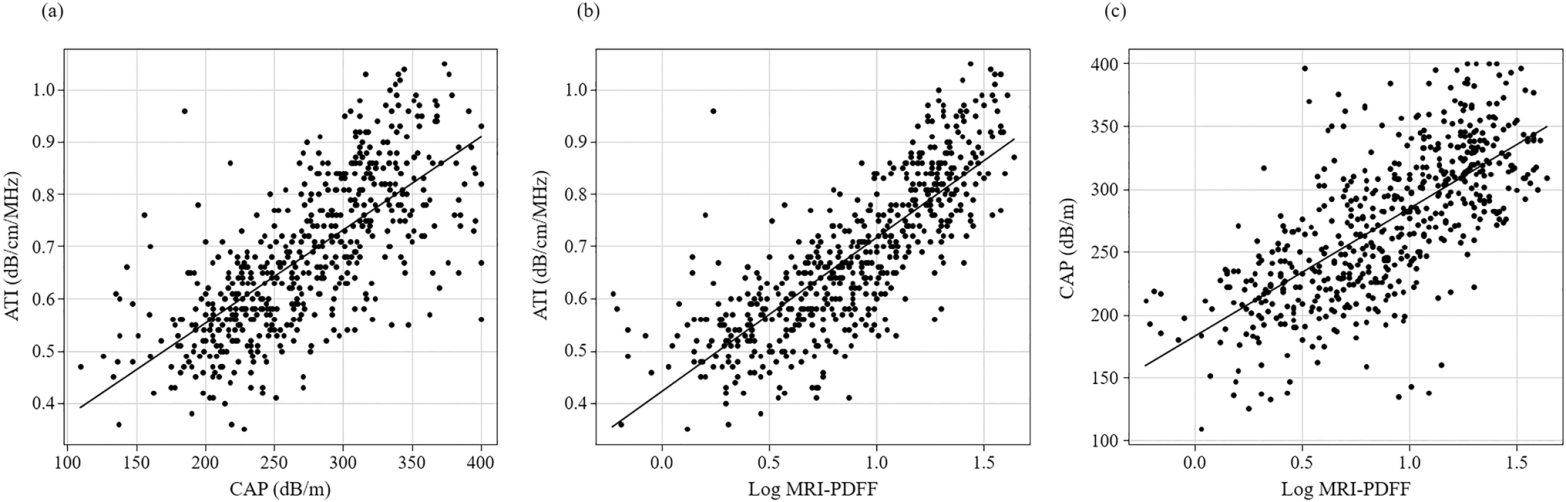
Correlations among ATI, CAP, and MRI-PDFF (*n* = 562). **a** Correlation between ATI and CAP. The *r* value was 0.707 (95% CI 0.663–0.746) (*P* < 0.0001). **b** Correlation between ATI and log MRI-PDFF. The *r* value was 0.799 (95% CI 0.767–0.827) (*P* < 0.0001). **c** Correlation between CAP and log MRI-PDFF. The *r* value was 0.694 (95% CI 0.648–0.734) (*P* < 0.0001)

Figure 2 shows the correlations between ATI, CAP, and MRI-PDFF. The CC for ATI and log MRI-PDFF showed strong correlation (*r* = 0.799). The CC value for CAP and log MRI-PDFF showed moderate correlation (*r* = 0.694). The CC for ATI and log MRI-PDFF was significantly higher than the CC for CAP and log MRI-PDFF (*P* < 0.001).

There were 53 patients (9.4%) with outliners of ATI values in the 90% predicted values analysis based on the correlation between MRI-PDFF values and ATI values (Supplementary Fig. 3). ATI values of 25 patients (group A) were higher than the upper limits of 90% predicted values and 28 patients (group C) were lower than the lower limits of 90% these values. The age, BMI, SCD, SWE and Fib-4 index in group A were not significantly difference in comparison with that of group B. The SCD in group C was significantly lower than that of group A and B (Supplementary Fig. 4). And there were 49 patients (8.7%) with outliners of CAP values in the 90% predicted values analysis based on the correlation between MRI-PDFF values and CAP values(Supplementary Fig. 5). CAP values of 28 patients (group D) were higher than the upper limits of 90% predicted values and 21 patients (group F) were lower than the lower limits of 90% these values. The BMI, SCD and SWE in group D were significantly higher than that of group E. The BMI and SCD in group F were significantly lower than that of group E (Supplementary Fig. 6).

### Correlations between ATI or CAP and log MRI-PDFF according to various parameters

We analyzed correlations between ATI or CAP and log MRI-PDFF according to various clinical parameters (Table 2). CCs for ATI and log MRI-PDFF in groups stratified by BMI (< 30, ≥ 30 kg/m^2^), SCD (< 25, ≥ 25 mm), 2D-SWE (< 1.8 m/s), FIB-4 index (≤ 2.67), ALBI score (< − 2.60), and type IV collagen 7 s (< 5.0 ng/ml) were significantly higher than those for CAP and log MRI-PDFF. Moreover, CCs for CAP and log MRI-PDFF in the BMI ≥ 30 kg/m^2^ groups were significantly lower than those in the BMI < 30 kg/m^2^ groups, respectively, but CCs for ATI and log MRI-PDFF were not. On the other hand, CCs for ATI and log MRI-PDFF in the 2D-SWE ≥ 1.8 m/s, FIB-4 index > 2.67, and ALBI score ≥ − 2.60 groups were significantly lower than those in the 2D-SWE < 1.8 m/s, FIB-4 index ≤ 2.67, and ALBI score < − 2.60 groups, respectively, but CCs for CAP and log MRI-PDFF were not. We also analyzed correlations between ATI or CAP values and log MRI-PDFF according to various clinical parameters in patients with MASLD (Supplementary Table 2). The results were essentially the same as results for the entire study cohort, but CCs for ATI and log MRI-PDFF in the BMI ≥ 30 kg/m^2^ groups were significantly lower than those in the BMI < 30 kg/m^2^ group.

**Table 2:** Correlations between attenuation imaging (ATI) or control attenuation parameter (CAP) and magnetic resonance imaging-based proton density fat fraction (MRI-PDFF) stratified by various parameters

Factor		ATI (95% confidence interval)	CAP (95% confidence interval)	*P*
All participants	(*n* = 562)	0.799 (0.767–0.827)	0.694 (0.648–0.734)	< 0.001
BMI (kg/m^2^)	< 30 (*n* = 431)	0.788 (0.750–0.822)	0.695 (0.642–0.741)	0.002
	≥ 30 (*n* = 131)	0.729 (0.637–0.800)	0.374 (0.216–0.512)	< 0.001
*P*		0.166	< 0.001	
SCD (mm)	< 25 (*n* = 489)	0.808 (0.775–0.837)	0.660 (0.607–0.708)	< 0.001
	≥ 25 (*n* = 73)	0.678 (0.531–0.786)	0.322 (0.099–0.514)	< 0.001
*P*		0.013	< 0.001	
2D-SWE (m/s)	< 1.8 (*n* = 482)	0.829 (0.798–0.855)	0.705 (0.657–0.747)	< 0.001
	≥ 1.8 (*n* = 79)	0.539 (0.361–0.679)	0.642 (0.490–0.756)	0.328
*P*		< 0.001	0.349	
FIB-4 index	≤ 2.67 (*n* = 432)	0.818 (0.784–0.847)	0.699 (0.647–0.744)	< 0.001
	> 2.67 (*n* = 127)	0.677 (0.570–0.761)	0.690 (0.494–0.713)	0.848
*P*		0.001	0.865	
ALBI score	< − 2.60 (*n* = 452)	0.804 (0.768–0.834)	0.703 (0.653–0.747)	< 0.001
	≥ − 2.60 (*n* = 104)	0.703 (0.590–0.789)	0.599 (0.459–0.710)	0.197
*P*		0.032	0.099	
Type IV collagen 7 s (ng/ml)	< 5.0 (*n* = 295)	0.829 (0.790–0.862)	0.731 (0.673–0.780)	0.002
	≥ 5.0 (*n* = 111)	0.719 (0.616–0.799)	0.590 (0.453–0.699)	0.094
*P*		0.013	0.025	

*ATI* attenuation imaging; *BMI* body mass index; *SCD* skin-to-liver capsule distance; *FIB-4* fibrosis-4; *ALBI* albumin-bilirubin

### ATI and CAP by liver steatosis grade with MRI-PDFF as the reference standard

**Fig. 3:**
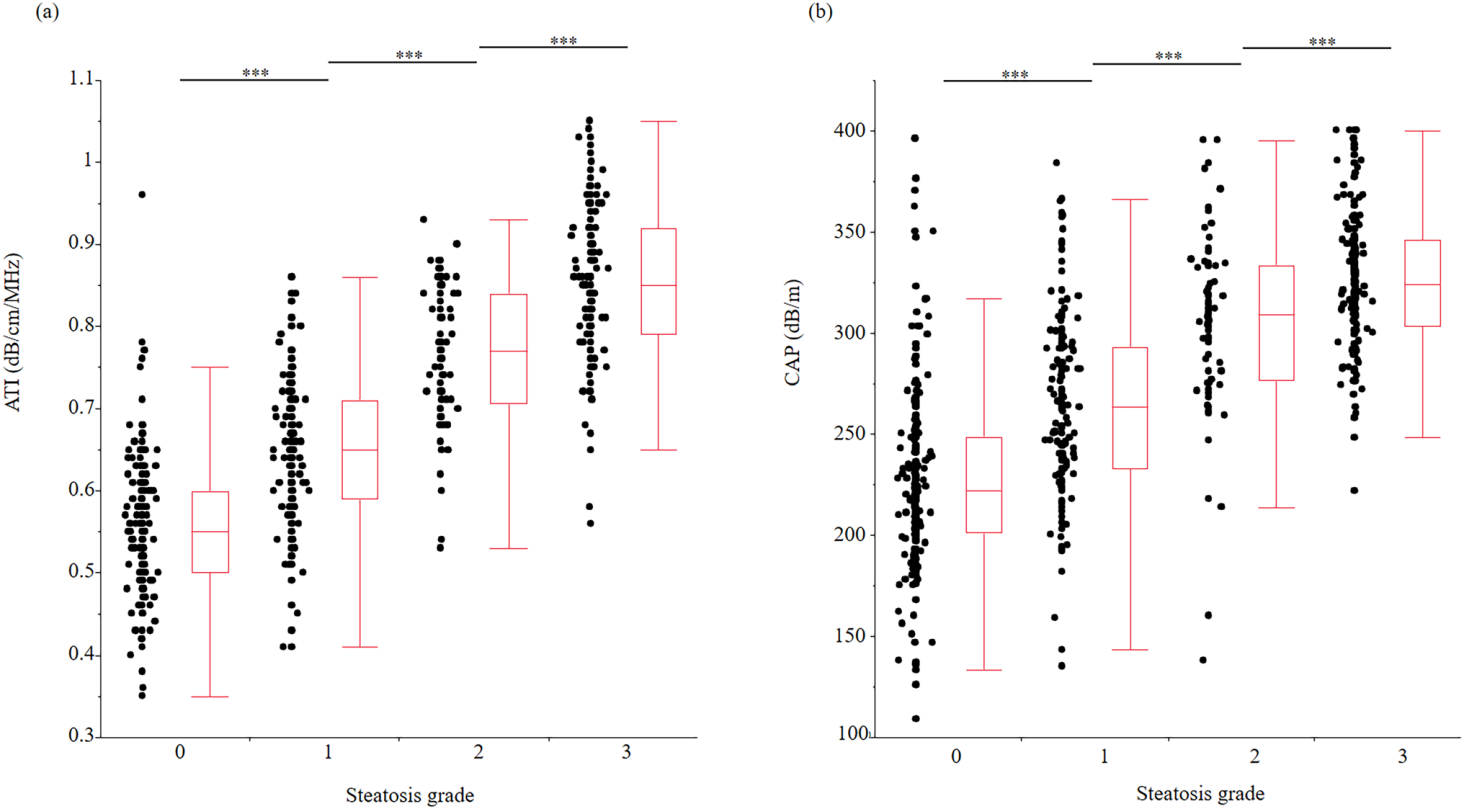
ATI and CAP (M/XL) values by steatosis grade with MRI-PDFF as the reference standard. **a** ATI values by steatosis grade with MRI-PDFF as the reference standard. Median ATI was 0.55 (interquartile range 0.50–0.60) dB/cm/MHz in patients with S0 steatosis (*n* = 185), 0.65 (0.59–0.71) dB/cm/MHz in patients with S1 steatosis (*n* = 159), 0.77 (0.71–0.84) dB/cm/MHz in patients with S2 steatosis (*n* = 77), and 0.85 (0.79–0.92) dB/cm/MHz in patients with S3 steatosis (*n* = 141). **b** CAP (M) values by steatosis grade with MRI-PDFF as the reference standard. Median CAP (M) was 222 (201–249) dB/m in patients with S0 steatosis (*n* = 185), 263 (233–293) dB/m in patients with S1 steatosis (*n* = 159), 309 (276–334) dB/m in patients with S2 steatosis (*n* = 77), and 324 (303–346) dB/m in patients with S3 steatosis (*n* = 141). *P < 0.05, **P < 0.01, ***P < 0.001

Figure 3 shows the relationship between steatosis grade with MRI-PDFF as the reference standard and ATI or CAP. ATI and CAP increased significantly with increasing steatosis grade. Essentially the same results were obtained on the basis of the degree of liver fibrosis (F0-2 vs F3-4) (Supplementary Fig. 7).

### Diagnostic performance of ATI and CAP according to steatosis grade with MRI-PDFF as the reference standard

**Fig. 4:**
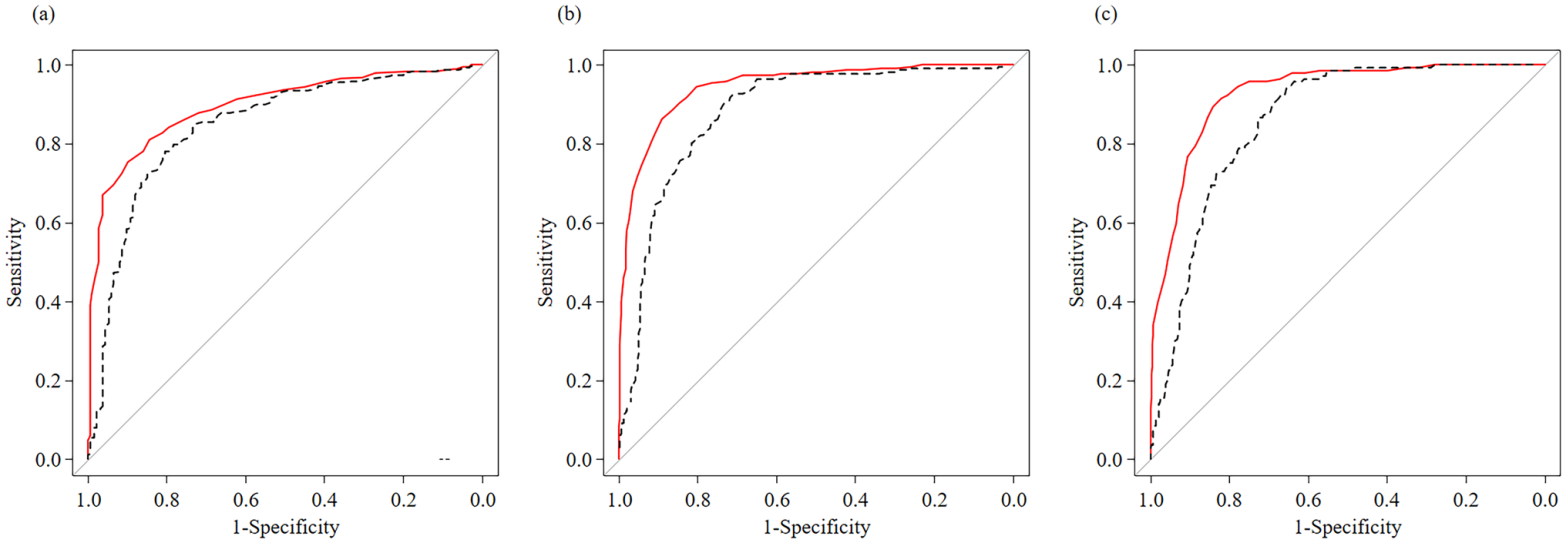
Receiver operating characteristic (ROC) curves and ATI and CAP cutoff levels for diagnosing hepatic steatosis grade based on MRI-PDFF as the reference standard. **a** Areas under the ROC and cutoff values for diagnosing hepatic steatosis grade ≥ S1 were 0.895 (95% CI 0.869–0.922) and 0.64 dB/cm/MHz for ATI (solid line) and 0.845 (95% CI 0.809–0.881) and 258 dB/m for CAP (M) (dotted line). **b** Areas under the ROC and cutoff values for diagnosing hepatic steatosis grade ≥ S2 were 0.944 (95% CI 0.926–0.963) and 0.72 dB/cm/MHz for ATI (solid line) and 0.881 (95% CI 0.852–0.910) and 272 dB/m for CAP (M) (dotted line). **c** Areas under the ROCs and cutoff values for diagnosing hepatic steatosis grade ≥ S3 were 0.928 (95% CI 0.906–0.950) and 0.75 dB/cm/MHz for ATI (solid line) and 0.860 (95% CI 0.829–0.890) and 276 dB/m for CAP (M) (dotted line)

AUROCs and cutoff values for ATI and CAP according to hepatic steatosis grade based on MRI-PDFF are shown in Fig. 4. The AUROC for ATI in detecting ≥ S1 steatosis was significantly higher than that for CAP (*P* = 0.004). The AUROC for ATI in detecting ≥ S2 steatosis was significantly higher than that for CAP (*P* < 0.001). The AUROC for ATI in detecting S3 steatosis was significantly higher than that for CAP (*P* < 0.001). In patients with MASLD, AUROCs for ATI in detecting ≥ S1, ≥ S2 and S3 steatosis were significantly higher than those for CAP (Supplementary Fig. 8). In patients with MASLD, the prevalence of hepatic steatosis (≥ S1) based on ATI and CAP cutoff values were 70.3% (242/344) and 72.1% (248/344), respectively. There were no statistically significant differences.

Table 3 shows the sensitivity, specificity, accuracy, positive predictive value (PPV), and negative predictive value (NPV). For detecting ≥ S1 hepatic steatosis, ATI did not have significantly higher sensitivity, specificity, accuracy, PPV, or NPV than CAP. By contrast, for detecting ≥ S2 and S3 hepatic steatosis, ATI had significantly higher specificity, accuracy, and PPV than CAP (all *P* < 0.001). The same analysis was performed in patients with MASLD (Supplementary Table 3). For detecting ≥ S1 hepatic steatosis, ATI had significantly higher specificity and PPV than CAP. (*P* = 0.007 and *P* = 0.011, respectively). Moreover, for detecting ≥ S2 and S3 hepatic steatosis, ATI had significantly higher sensitivity, accuracy, and NPV than CAP.

**Table 3:** Diagnostic performance of attenuation imaging (ATI) and control attenuation parameter (CAP) in detecting S1, S2, and S3 steatosis

	Cutoff value	Sensitivity (%)	Specificity (%)	Accuracy (%)	PPV (%)	NPV (%)
S1 (≥ 5% hepatic steatosis)						
CAP (dB/m)	258	78.0	80.5	78.8	89.1	64.2
ATI (dB/cm/MHz)	0.64	80.9	84.3	82.0	91.3	68.4
*P*		0.368	0.413	0.201	0.362	0.375
S2 (≥ 33% hepatic steatosis)						
CAP (dB/m)	272	90.4	73.3	79.9	68.2	92.3
ATI (dB/cm/MHz)	0.72	86.2	89.0	87.9	83.2	91.1
*P*		0.233	< 0.001	< 0.001	< 0.001	0.660
S3 (> 66% hepatic steatosis)						
CAP (dB/m)	276	94.3	65.3	72.6	47.7	97.2
ATI (dB/cm/MHz)	0.75	89.4	84.1	85.4	65.3	95.9
*P*		0.377	< 0.001	< 0.001	< 0.001	0.522

Steatosis grade: S0, MRI-PDFF < 5.2%; S1, 5.2% ≤ MRI-PDFF < 11.3%; S2, 11.3% ≤ MRI-PDFF < 17.1%; and S3, MRI-PDFF ≥ 17.1%

S0, S1, S2, and S3 correspond to histologic hepatic steatosis grades of < 5%, 5–33%, 34–66%, and > 66%, respectively

*PPV* positive predictive value; *NPV* negative predictive value

## Discussion

In this prospective large multicenter cohort study based on over 500 examinations, ATI and CAP were highly accurate in diagnosing hepatic steatosis with MRI-PDFF as the reference standard. Both ATI and CAP increased significantly with the progression of steatosis grade independent of the degree of liver fibrosis. However, the CC for ATI and MRI-PDFF was significantly higher than the CC for CAP and MRI-PDFF. Moreover, ATI was not affected by BMI. In addition, AUROCs for ATI in detecting ≥ S1, S2, or S3 hepatic steatosis were significantly higher than those for CAP. ATI had significantly higher specificity, accuracy, PPV, and NPV for detecting ≥ S2 or S3 hepatic steatosis than CAP. As a result, the diagnostic performance of ATI is superior to that of CAP. Essentially the same results were obtained for the patients with MASLD.

MRI-PDFF is the non-invasive method for diagnosing hepatic steatosis grade with the highest diagnostic performance [20]. However, MRI-PDFF has several disadvantages including cost and lower patient acceptance. On the other hand, ATI and CAP are non-invasive methods for diagnosing hepatic steatosis that are cost-effective and have good patient acceptance. Moreover, the correlation between ATI and MRI-PDFF was strong. Therefore, ATI can be considered a non-invasive alternative to MRI-PDFF. Based on the perspective of outliners, patients with low SCD tended to have lower ATI values. CAP values of patients with high BMI, SCD, and SWE values tended to be higher and CAP values of patients with low BMI and SCD tended to be lower. This should be noted.

CAP has been widely used as an attenuation imaging method for diagnosing non-invasive hepatic steatosis, facilitating swift bedside assessments of tissue stiffness. Its usefulness has been widely reported. However, a study by Victor de Ledinghen et al. revealed a 7.7% failure rate among 5325 CAP examinations. Measurement failure was associated with female gender, higher BMI, and the presence of metabolic syndrome. Elevated CAP values were observed in patients with BMI ≥ 25 kg/m^2^, metabolic syndrome, alcohol consumption > 14 drinks per week, or liver stiffness > 6 kPa [29]. In this study, the CC for CAP and MRI-PDFF was significantly lower in patients with BMI ≥ 30 kg/m^2^ than in patients with BMI < 30 kg/m^2^. Moreover, despite using the XL probe for patients with SCD ≥ 25 mm, the CC for CAP and MRI-PDFF was significantly lower than that of ATI and the CC in patients with SCD ≥ 25 mm significantly lower than that in patients with SCD < 25 mm. This result suggested that CAP is affected by BMI or SCD despite using the XL probe in patients with SCD ≥ 25 mm. The American Association for The Study of Liver Disease (AASLD) Practice Guidance on the Clinical Assessment and Management of Nonalcoholic Fatty Liver Disease states that CAP does not accurately quantify or monitor changes in liver fat [30].

Regarding the influence of hepatic fibrosis on ATI and CAP, Yuri et al. reported that ATI is not affected by liver fibrosis [31]. On the other hand, the CCs between ATI and MRI-PDFF in patients with high 2D-SWE, high FIB-4 index, or high type IV collagen 7 s, which reflect advanced hepatic fibrosis, were significantly lower than those in patients with low SWE, low FIB-4 index, or low type IV collagen 7 s. However, the CCs for CAP and MRI-PDFF were not significantly different. Therefore, the influence of ATI on the diagnosis of hepatic steatosis in patients with advanced liver fibrosis should be kept in mind.

The AUROC for diagnosing ≥ S1 hepatic steatosis using ATI has been reported to be more than 0.85 [12, 32–35]. In this study, the AUROC for diagnosing ≥ S1 hepatic steatosis using ATI was 0.895, which was higher than using CAP. Ferraioli et al. have investigated the diagnostic ability of ATI and CAP [17]. In their study, ATI was more accurate than CAP for detecting and quantifying liver steatosis with a statistically significant difference for only ≥ S2. However, in our study, the AUROC for ATI in diagnosing all hepatic steatosis grades (≥ S1, ≥ S2, and S3) was significantly higher than for CAP. The specificity, accuracy, and PPV for ATI in detecting ≥ S2 or S3 hepatic steatosis were significantly higher than those for CAP. This result means that for detecting ≥ S2 or S3 hepatic steatosis grade, there are fewer false positives. Accordingly, ATI is considered more useful than CAP in diagnosing hepatic steatosis. Particularly, ATI had high accuracy for detecting ≥ S2 hepatic steatosis. The PPVs of ATI and CAP decreased 10–30% with the progression of steatosis grade. Of note, the PPVs of ATI and CAP in patients with S3 steatosis were lower than those in patients with S1 or S2 steatosis. Lower prevalence of subjects is known to be associated with lower PPV. The prevalence of patients with S3 steatosis was significantly lower than that in S1 and S2 steatosis. Moreover, the prevalence of patients with S2 steatosis was significantly lower than in S1 steatosis, which might be one reason why PPV was low in S3 steatosis.

In this prospective study, the CAP cutoff values for diagnosing ≥ S1, S2, or S3 hepatic steatosis were 258 dB/m, 272 dB/m, and 276 dB/m, respectively. The cutoff values for CAP were only slightly higher or slightly lower than the values in a meta-analysis [36]. The ATI cutoff values for diagnosing ≥ S1, S2, or S3 hepatic steatosis grade were 0.64 dB/cm/MHz, 0.72 dB/cm/MHz, and 0.75 dB/cm/MHz, respectively. The cutoff value for ≥ S1 in this study was almost the same as that in a pilot study; the cutoff value for ≥ S2 was the same value [32]. Therefore, the ATI cutoff values for detecting each steatosis grade in this prospective and multicenter study seem to be more applicable for use in daily practice.

In some of MASLD patients, ATI and CAP values have been less than the cutoff values for S1. These patients have been initially diagnosed MASLD by MRI-PDFF, liver biopsy and ultrasound including attenuation imaging before this research start. Therefore, some of the MASLD patients with the improvement of hepatic steatosis had the value less than cutoff values of S1. And SWE value in MASLD patients diagnosed as S0 based on ATI and CAP cutoff value was not higher than that of S1-3 (Supplementary Fig. 9). Therefore, ATI and CAP in the MASLD patients with S0 was not related with advance hepatic fibrosis.

Our study had several limitations. First, patients with various etiologies of liver disease were included. However, SLD consists of patients with not only MASLD but also other etiologies of hepatic steatosis. Therefore, the results of this study are useful in the clinical setting. Second, ATI, CAP and MRI-PDFF measurements were performed within 1 month. Therefore, steatosis grade could have decreased because of diet and exercise therapy. However, we think that there were few patients whose steatosis grade decreased over only 1 month in the clinical setting. Third, almost all subjects were Japanese. Further study of patients of various races is warranted.

In conclusion, ATI was strongly correlated with MRI-PDFF and had good diagnostic ability for each hepatic steatosis grade in this multicenter prospective study. In addition, ATI is a better non-invasive method for diagnosing hepatic steatosis than CAP. In particular, ATI is less affected by body shape. Further prospective studies should be performed throughout the world.

## Supplementary Information

Below is the link to the electronic supplementary material.

Supplementary file1 (DOCX 19 KB)

Supplementary file2 (DOCX 20 KB)

Supplementary file3 (DOCX 18 KB)

## Abbreviations

ATI: Attenuation imaging
CAP: Controlled attenuation parameter
MRI-PDFF: Magnetic resonance imaging-based proton density fat fraction
HCC: Hepatocellular carcinoma
ROI: Region of interest
AUROC: Area under the receiver operating characteristic curve
IQR: Interquartile range
2D-SWE: 2-Dimentional shear wave elastography
SCD: Skin-to-liver capsule distance
CC: Correlation coefficient
BMI: Body mass index
HBV: Hepatitis B virus
HCV: Hepatitis C virus
MASLD: Metabolic associated steatotic liver disease
AIH: Autoimmune hepatitis
PBC: Primary biliary cholangitis
PPV: Positive predictive value
NPV: Negative predictive value

## References

[1] Rinella ME, Lazarus JV, Ratziu V, et al. A multisociety Delphi consensus statement on new fatty liver disease nomenclature. J Hepatol. 2023;79:1542–56. 37364790 10.1016/j.jhep.2023.06.003

[2] Angulo P, Kleiner DE, Dam-Larsen S, et al. Liver fibrosis, but no other histologic features, associates with long-term outcomes of patients with nonalcoholic fatty liver disease. Gastroenterology. 2015;149:389–97. 25935633 10.1053/j.gastro.2015.04.043 PMC4516664

[3] Arulanandan A, Ang B, Bettencourt R, et al. Association between quantity of liver fat and cardiovascular risk in patients with nonalcoholic fatty liver disease independent of nonalcoholic steatohepatitis. Clin Gastroenterol Hepatol. 2015;13:1513–20. 25661453 10.1016/j.cgh.2015.01.027

[4] Nasr P, Fredrikson M, Ekstedt M, et al. The amount of liver fat predicts mortality and development of type 2 diabetes in non-alcoholic fatty liver disease. Liver Int. 2020;40:1069–78. 32087038 10.1111/liv.14414

[5] Leandro G, Mangia A, Hui J, et al. Relationship between steatosis, inflammation, and fibrosis in chronic hepatitis C: a meta-analysis of individual patient data. Gastroenterology. 2006;130:1636–42. 16697727 10.1053/j.gastro.2006.03.014

[6] Sano T, Amano K, Ide T et al. Metabolic management after sustained virologic response in elderly patients with hepatitis C virus: a multicenter study. Hepatol Res. 2024; 54; 326–335 10.1111/hepr.13993 37975277

[7] Mak LY, Hui RW, Fung J, et al. Reduced hepatic steatosis is associated with higher risk of hepatocellular carcinoma in chronic hepatitis B infection. Hepatol Int. 2021;15:901–11. 34152534 10.1007/s12072-021-10218-2

[8] Shimose S, Hiraoka A, Casadei-Gardini A, et al. The beneficial impact of metabolic dusfunction associated fatty liver disease on lenvatinib treatment in patients with non-viral hepatocellular carcinoma. Hepatol Res. 2023;53:104–15. 36149726 10.1111/hepr.13843

[9] Reeder SB, Robson PM, Yu H, et al. Quantification of hepatic steatosis with MRI: the effects of accurate fat spectral modeling. J Magn Reson Imaging. 2009;29:1332–9. 19472390 10.1002/jmri.21751 PMC2689318

[10] Permutt Z, Le TA, Peterson MR, et al. Correlation between liver histology and novel magnetic resonance imaging in adult patients with non-alcoholic fatty liver disease -MRI accurately quantifies hepatic steatosis in NAFLD. Aliment Pharmacol Ther. 2012;36:22–9. 22554256 10.1111/j.1365-2036.2012.05121.x PMC3437221

[11] Ferraioli G. Quantitative assessment of liver steatosis using ultrasound controlled attenuation parameter (Echosens). J Med Ultrason. 2001;2021(48):489–95. 10.1007/s10396-021-01106-1 PMC8578057 34132934

[12] Tada T, Iijima H, Kobayashi N, et al. Usefulness of attenuation imaging with an ultrasound scanner for the evaluation of hepatic steatosis. Ultrasound Med Bio. 2019;45:2679–87. 31277922 10.1016/j.ultrasmedbio.2019.05.033

[13] Fujiwara Y, Kuroda H, Abe T, et al. The B-mode image-guided ultrasound attenuation parameter accuracy detects hepatic steatosis in chronic liver disease. Ultrasound Med Bio. 2018;44:2223–32. 30077415 10.1016/j.ultrasmedbio.2018.06.017

[14] Tada T, Kumada T, Toyoda H, et al. Utility of attenuation coefficient measurement using an ultrasound-guided attenuation parameter forevaluation of hepatic steatosis: comparison with MRI-determined proton density fat fraction. Am J Roentgenol. 2019;212:332–41. 30476453 10.2214/AJR.18.20123

[15] Tamaki N, Koizumi Y, Hirooka M, et al. Novel quantitative assessment system of liver steatosis using a newly developed attenuation measurement method. Hepatol Res. 2018;48:821–8. 29679473 10.1111/hepr.13179

[16] Ferraioli G, Raimondi A, Maiocchi L, et al. Quantification of liver fat content with the iATT algorithm: correlation with controlled attenuation parameter. Diagnostics (Basel). 2022;12:1787. 35892497 10.3390/diagnostics12081787 PMC9394249

[17] Ferraioli G, Maiocchi L, Raciti MV, et al. Detection of liver steatosis with a novel ultrasound- based technique: a pilot study using MRI-derived proton density fat fraction as the gold standard. ClinTransl Gastroenterol. 2019;10: e00081. 10.14309/ctg.0000000000000081 PMC6884349 31609745

[18] Barre RG, Wilson SR, Rubens D, et al. Update to the society of radiologists in ultrasound liver elastography consensus statement. Radiology. 2020;296:263–74. 32515681 10.1148/radiol.2020192437

[19] Castera L, Forns X, Alberti A. Non-invasive evaluation of liver fibrosis using transient elastography. J Hepatol. 2008;48:835–47. 18334275 10.1016/j.jhep.2008.02.008

[20] Imajo K, Kessoku T, Honda Y, et al. Magnetic resonance imaging more accurately classifies steatosis and fibrosis in patients with nonalcoholic fatty liver disease than transient elastography. Gastroenterology. 2016;150:626–37. 26677985 10.1053/j.gastro.2015.11.048

[21] Younossi ZM, Stepanova M, Rafiq N, et al. Pathologic criteria for nonalcoholic steatohepatitis: interprotocol agreement and ability to predict liver-related mortality. Hepatology. 2011;53:1874–82. 21360720 10.1002/hep.24268

[22] Shah AG, Lydecker A, Murray K, et al. Comparison of noninvasive markers of fibrosis in patients with nonalcoholic fatty liver disease. Clin Gastroenterol Hepatol. 2009;7:1104–12. 19523535 10.1016/j.cgh.2009.05.033 PMC3079239

[23] Johnson PJ, Berhane S, Kagebayashi C, et al. Assessment of liver function in patients with hepatocellular carcinoma: a new evidence-based approach-the ALBI grade. J Clin Oncol. 2015;33:550–8. 25512453 10.1200/JCO.2014.57.9151 PMC4322258

[24] Sumida Y, Yoneda M, Hyogo H, et al. A simple clinical scoring system using ferritin, fasting insulin and type IV collagen 7S for predicting steatohepatitis in nonalcoholic fatty liver disease. J Gastroenterol. 2011;46:257–68. 20842510 10.1007/s00535-010-0305-6

[25] World Health Organization. Overweight and Obesity. https://www.who.int/news-room/fact-sheets/detail/obesity-and-overweight

[26] Jeon UK, Lee JM, Joo I, et al. Prospective evaluation of hepatic steatosis using ultrasound attenuation imaging in patients with chronic liver disease with magnetis resonance imaging proton density fat fraction as the reference standard. Ultrasound Med Biol. 2019;45:1407–16. 30975533 10.1016/j.ultrasmedbio.2019.02.008

[27] Barr GR. Shear wave liver elastography. Abdom Radiol (NY). 2018;43:800–7. 29116341 10.1007/s00261-017-1375-1

[28] Guilford JP. Fundamental statistics in psychology and education. New York: McGraw Hill; 1956.

[29] de Ledinghen V, Vergniol J, Capdepont M, et al. Controlled attenuation parameter (CAP) for the diagnosis of steatosis: a prospective study of 5323 examinations. J Hepatol. 2014;60:1026–31. 24378529 10.1016/j.jhep.2013.12.018

[30] Rinella ME, Neuschwander-Tetri BA, Siddiqui MS, et al. AASLD practice guidance on the clinical assessment and management of nonalcoholic fatty liver disease. Hepatology. 2023;77:1797–835. 36727674 10.1097/HEP.0000000000000323 PMC10735173

[31] Yuri M, Nishimura T, Tada T, et al. Diagnosis of hepatic steatosis based on ultrasound attenuation imaging is not influenced by liver fibrosis. Hepatol Res. 2022;52:1009–19. 36018852 10.1111/hepr.13831

[32] Ferraioli G, Maiocchi M, Raciti MV, et al. Detection of liver steatosis with a novel ultrasound- based technique: a pilot study using MRI-derived proton density fat fraction as the gold standard clinical and translational. Gastroenterology. 2019;10: e00081. 10.14309/ctg.0000000000000081 PMC6884349 31609745

[33] Lee DH, Cho EJ, Bae JS, et al. Accuracy of two-dimensional shear wave elastography and attenuation imaging for evaluation of patients with nonalcoholic steatohepatitis. Clin Gastroenterol Hepatol. 2021;19:797–805. 32450363 10.1016/j.cgh.2020.05.034

[34] Jang JK, Lee ES, Seo JW, et al. Two dimensional Shear-Wave elastography and US attenuation imaging for nonalcoholic steatohepatitis diagnosis: a cross-sectional. Multicenter Study Radiology. 2022;305:118–26. 35727151 10.1148/radiol.220220

[35] Sugimoto K, Moriyasu F, Oshiro H, et al. The role of multiparametric US of the liver for the evaluation of nonalcoholic steatohepatitis. Radiology. 2020;296:532–40. 32573385 10.1148/radiol.2020192665

[36] Karlas T, Petroff D, Sasso M, et al. Individual patient data meta-analysis of controlled attenuation parameter (CAP) technology for assessing steatosis. J Hepatol. 2017;66:1022–30. 28039099 10.1016/j.jhep.2016.12.022

