# Supplementary material for: Diagnostic performance of attenuation imaging versus controlled attenuation parameter for hepatic steatosis with MRI-based proton density fat fraction as the reference standard: a prospective multicenter study
Source: J Gastroenterol. 2025 Feb 24;60(6):727–37. doi: 10.1007/s00535-025-02224-0 (PMC12095409; doi:10.1007/s00535-025-02224-0)
Supplement: Supplementary file 1 — Supplementary file1 (DOCX 19 KB) [file 535_2025_2224_MOESM1_ESM.docx]

**Philips**

Magnetic resonance imaging (MRI) was performed using a 3T system (Ingenia; Philips Healthcare, Best, Netherlands). With Ingenia, proton density fat fraction (PDFF) was measured on the basis of a chemical shift encoded technique with modified Dixon quantification (mDIXON-Quant) within 1 month of ATI measurement. Parameters for PDFF were as follows: repetition time, 5.7 ms; echo time (TE) / delta TE, 0.98/0.7 ms; 6 echoes; field of view, 40 cm; matrix, 160 × 160; slice thickness, 6.0 mm; acquisition time, single breath-hold of 16 s; phase field of view, 0.87; flip angle, 3°; NSA, 1; bandwidth, 2,367.4 Hz; SENSE factor, phase 2; and number of slices, 77.

**GE**

MRI was performed using a 3T system (Discovery MR750w 3.0 T; GE Healthcare, Waukesha, WI). With Discovery MR 750w, PDFF was measured using a multiecho Dixon method (IDEAL-IQ sequence) within 1 month of ATI measurement. Parameters for PDFF were as follows: repetition time, 6.6 ms; echo time (TE) /delta TE, 0.98/0.7 ms; echo time, 6 echoes, ranging from 0.9 to 4.8 ms; field of view, 44 cm; matrix, 160 × 160; slice thickness, 8.0 mm; acquisition time, single breath-hold of 19 s; phase field of view, 0.80; flip angle, 1°; echo train length, 3; NEX, 0.50; bandwidth, 111.11 kHz; and acceleration factor, phase 2.50 slice 1.00.

**Siemens**

MRI was performed using a 3T system (Skyra; Siemens Healthcare, Munich, Germany) with a 32-channel phased-array coil. MRI-PDFF data were obtained using the multi-echo Dixon method with the following parameters: repetition time: 9 ms; echo time: 1.05, 2.46, 3.69, 4.92, 6.15, and 7.38 ms; flip angle: 4°; matrix size, 320 × 280; axial imaging plane; section thickness, 6 mm; field of view (FOV), 450 mm; fractional phase FOV, ranging from 0.75 to 1; 1 signal acquired; bandwidth, 1,080 Hz/Px; and imaging time consisting of two breath holds (approximately 16 s each). In proximity to the regions of interest (ROIs) drawn for LSM, new ROIs were drawn on both the in-phase and out-of-phase images for PDFF measurements.
