# Supplementary material for: Diagnostic performance of attenuation imaging versus controlled attenuation parameter for hepatic steatosis with MRI-based proton density fat fraction as the reference standard: a prospective multicenter study
Source: J Gastroenterol. 2025 Feb 24;60(6):727–37. doi: 10.1007/s00535-025-02224-0 (PMC12095409; doi:10.1007/s00535-025-02224-0)
Supplement: Supplementary file 2 — Supplementary file2 (DOCX 20 KB) [file 535_2025_2224_MOESM2_ESM.docx]

Supplementary Table 2. Correlations between attenuation imaging (ATI) or control attenuation parameter (CAP) and magnetic resonance imaging-based proton density fat fraction (MRI-PDFF) stratified by various parameters in MASLD patients.

| Factor |  | | ATI (95% confidence interval) | CAP (95% confidence interval) | *P* |
| --- | --- | --- | --- | --- | --- |
| All participants | | (n=344) | 0.809 (0.769–0.842) | 0.583 (0.509–0.649) | <0.001 |
| BMI (kg/m^2^) | <30 (n=226) | | 0.831(0.786–0.868) | 0.638 (0.554–0.710) | <0.001 |
|  | ≥30 (n=118) | | 0.707 (0.603–0.787) | 0.364 (0.196–0.511) | <0.001 |
| *P* |  | | < 0.001 | < 0.001 |  |
| SCD (mm) | <25 (n=281) | | 0.827 (0.786–0.861) | 0.638 (0.563–0.702) | <0.001 |
|  | ≥25 (n=63) | | 0.632 (0.456–0.761) | 0.231 (-0.018–0.453) | <0.001 |
| *P* |  | | < 0.001 | < 0.001 |  |
| 2D-SWE (m/s) | <1.8 (n=297) | | 0.825 (0.784–0.858) | 0.612 (0.536–0.679) | <0.001 |
|  | ≥1.8 (n=46) | | 0.635 (0.422–0.781) | 0.486 (0.228–0.680) | 0.151 |
| *P* |  | | 0.006 | 0.235 |  |
| FIB-4 index | ≤2.67 (n=285) | | 0.770 (0.718–0.814) | 0.540 (0.453–0.618) | 0.044 |
|  | >2.67 (n=59) | | 0.749 (0.610–0.844) | 0.484 (0.260–0.658) | 0.740 |
| *P* |  | | 0.710 | <0.001 |  |
| ALBI score | <-2.60 (n=297) | | 0.815(0.773–0.850) | 0.606 (0.529–0.674) | <0.001 |
|  | ≥-2.60 (n=47) | | 0.679 (0.487–0.809) | 0.466 (0.206–0.664) | 0.033 |
| *P* |  | | 0.037 | 0.190 |  |
| Type IV collagen 7s (ng/mL) | <5.0 (n=207) | | 0.832 (0.784–0.869) | 0.662 (0.578–0.732) | <0.001 |
|  | ≥5.0 (n=66) | | 0.757 (0.630–0.844) | 0.534(0.335–0.687) | 0.375 |
| *P* |  | | <0.001 | 0.375 |  |

ATI. Attenuation Imaging; BMI, body mass index; SCD, skin-to-liver capsule distance; FIB-4, fibrosis-4; ALBI, albumin-bilirubin.
