# Supplementary material for: Diagnostic performance of attenuation imaging versus controlled attenuation parameter for hepatic steatosis with MRI-based proton density fat fraction as the reference standard: a prospective multicenter study
Source: J Gastroenterol. 2025 Feb 24;60(6):727–37. doi: 10.1007/s00535-025-02224-0 (PMC12095409; doi:10.1007/s00535-025-02224-0)
Supplement: Supplementary file 3 — Supplementary file3 (DOCX 18 KB) [file 535_2025_2224_MOESM3_ESM.docx]

Supplementary Table 3. Diagnostic performance of attenuation imaging (ATI) and control attenuation parameter (CAP) in detecting S1, S2, and S3 steatosis in MAFLD patients(n=344)

|  | Cutoff value | Sensitivity (%) | Specificity (%) | Accuracy (%) | PPV (%) | NPV (%) |
| --- | --- | --- | --- | --- | --- | --- |
| S1 (≥ 5% hepatic steatosis) |  |  |  |  |  |  |
| CAP (dB/m) | 268 | 80.1 | 73.1 | 79.1 | 94.4 | 39.6 |
| ATI (dB/cm/MHz) | 0.67 | 81.8 | 94.2 | 83.7 | 98.8 | 48.0 |
| *P* |  | 0.673 | 0.007 | 0.141 | 0.011 | 0.254 |
| S2 (≥ 33% hepatic steatosis) |  |  |  |  |  |  |
| CAP (dB/m) | 304 | 71.6 | 83.0 | 76.5 | 84.9 | 68.5 |
| ATI (dB/cm/MHz) | 0.73 | 84.8 | 87.1 | 85.8 | 89.8 | 81.0 |
| *P* |  | 0.002 | 0.414 | 0.002 | 0.197 | 0.012 |
| S3 (> 66% hepatic steatosis) |  |  |  |  |  |  |
| CAP (dB/m) | 311 | 71.4 | 75.4 | 73.8 | 64.6 | 80.7 |
| ATI (dB/cm/MHz) | 0.76 | 88.7 | 79.1 | 82.8 | 72.8 | 91.8 |
| *P* |  | <0.001 | 0.416 | 0.005 | 0.140 | 0.003 |

Steatosis grade: S0, MRI-PDFF < 5.2%; S1, 5.2% ≤ MRI-PDFF < 11.3%; S2, 11.3% ≤ MRI-PDFF < 17.1%; and S3, MRI-PDFF ≥ 17.1%.

S0, S1, S2, and S3 correspond to histologic hepatic steatosis grades of <5%, 5–33%, 34–66%, and >66%, respectively.

PPV, positive predictive value; NPV, negative predictive value.
